# Mitigating Antibiotic Resistance: The Utilization of CRISPR Technology in Detection

**DOI:** 10.3390/bios14120633

**Published:** 2024-12-20

**Authors:** Xuejiao Zhang, Zhaojie Huang, Yanxia Zhang, Wen Wang, Zihong Ye, Pei Liang, Kai Sun, Wencheng Kang, Qiao Tang, Xiaoping Yu

**Affiliations:** 1 Key Laboratory of Microbiological Metrology, Measurement & Bio-product Quality Security, State Administration for Market Regulation, College of Life Science, China Jiliang University, Hangzhou 310018, China; p23091055079@cjlu.edu.cn (X.Z.); s23090710016@cjlu.edu.cn (Z.H.); s24090710073@cjlu.edu.cn (Y.Z.); wangwen@cjlu.edu.cn (W.W.); zhye@cjlu.edu.cn (Z.Y.); sunkai@cjlu.edu.cn (K.S.); 2 College of Optical and Electronic Technology, China Jiliang University, Hangzhou 310018, China; plianghust@cjlu.edu.cn; 3 Inner Mongolia Institute of Metrology and Testing, Hohhot 010030, China; kangwc324@163.com; 4 Zhejiang Provincial Key Laboratory of Biometrology and Inspection & Quarantine, China Jiliang University, Hangzhou 310018, China

**Keywords:** CRISPR, antibiotic detection, nucleic acid targets, non-nucleic acid targets

## Abstract

Antibiotics, celebrated as some of the most significant pharmaceutical breakthroughs in medical history, are capable of eliminating or inhibiting bacterial growth, offering a primary defense against a wide array of bacterial infections. However, the rise in antimicrobial resistance (AMR), driven by the widespread use of antibiotics, has evolved into a widespread and ominous threat to global public health. Thus, the creation of efficient methods for detecting resistance genes and antibiotics is imperative for ensuring food safety and safeguarding human health. The clustered regularly interspaced short palindromic repeats (CRISPR) and CRISPR-associated proteins (Cas) systems, initially recognized as an adaptive immune defense mechanism in bacteria and archaea, have unveiled their profound potential in sensor detection, transcending their notable gene-editing applications. CRISPR/Cas technology employs Cas enzymes and guides RNA to selectively target and cleave specific DNA or RNA sequences. This review offers an extensive examination of CRISPR/Cas systems, highlighting their unique attributes and applications in antibiotic detection. It outlines the current utilization and progress of the CRISPR/Cas toolkit for identifying both nucleic acid (resistance genes) and non-nucleic acid (antibiotic micromolecules) targets within the field of antibiotic detection. In addition, it examines the current challenges, such as sensitivity and specificity, and future opportunities, including the development of point-of-care diagnostics, providing strategic insights to facilitate the curbing and oversight of antibiotic-resistance proliferation.

## 1. Introduction

### 1.1. Antibiotics

Antibiotics are mainly used to treat a range of bacterially induced bacterial infections. They are undoubtedly one of the most effective medications ever found in the history of human drug therapy. Most antibiotic chemical scaffolds currently in clinical use, such as β-lactams (penicillins and cephalosporins), macrolides, aminoglycosides, tetracyclines, glycopeptides, and fluoroquinolones, were discovered by scientists during the two decades between the clinical use of penicillin in the early 1940s and the discovery of fluoroquinolone antibiotics in 1962 [1]. Currently, in addition to their widespread use as therapeutic agents in humans, livestock, and aquaculture, antibiotics are also used as feed additives in animal husbandry. The use of antibiotic drugs has dramatically reduced the mortality rate caused by bacterial infections. However, the overuse of antibiotics has been recognized as a serious global public health problem due to the increase in the number of antibiotic-resistant bacteria in recent years, the decrease in the efficacy of antibiotics, and the multiplicity of infections [2].

Earlier this year, Chen et al. released a research survey showing that total antibiotic use in the Yangtze River Basin alone exceeded 1600 tons/year between 2013 and 2021 [3]. Grand View Research, a market research firm, recently published a market study on the antibiotics market, showing a global antimicrobial additives market size of USD 3.11 billion in 2023 [4], with increasing consumption of pharmaceuticals and medical devices and other end-use industries such as healthcare, personal care, and electronics, demand for antibiotics is growing at a significant rate. The global antimicrobial additive market size is expected to reach an estimated USD 5.63 billion by 2030 [4].

Antibiotics cannot be completely metabolized in living organisms and are often excreted into the environment as parent compounds or metabolites. Due to the limited removal capacity of conventional wastewater-treatment processes, antibiotics are continuously introduced into the environment, causing environmental pollution [5]. The most significant consequence of antibiotic release in the natural environment is the generation of antibiotic-resistance genes (ARGs) and -resistant bacteria (ARB), which not only causes pollution of the natural environment but also disrupt the structure and functionality of environmental microbial communities, with possible implications for human health and the evolution of ecological microbial populations [6].

Globally, several hundreds of thousands of people die each year from antibiotic-resistant bacteria [7], and it is estimated that by 2050, the global economic cost of ARB infections will reach USD 100 trillion [8]. In 2008, Rice et al. identified six “antibiotic failure” pathogens [9], which, according to a report published by the World Health Organization (WHO), can be considered the most common pathogens. According to the WHO 2024 Catalogue of Priority Bacterial Pathogens [10], a total of 15 drug-resistant bacteria are listed, and the existence of these drug-resistant bacteria poses a serious threat to global public health. It requires practical public health approaches and new antibiotic research to deal with it. However, it highlights that the development of new antibiotics is significantly outpaced by the evolution of bacterial resistance [11]. In the 21st century, only a handful of new antibiotic classes are available for clinical use. Without new antibiotics, by 2050, the number of deaths from drug-resistant infections is expected to reach 10 million per year globally [12].

Antibiotic resistance has become one of the most significant challenges [8]. A cross-national analysis of studies published in 2022 showed that high levels of resistance to pathogens are strongly associated with high mortality rates due to these pathogens [7]. Furthermore, the evolution and spread of antimicrobial resistance have been accelerated by the epidemic of pneumococcal pneumonia [13], and bacterial resistance poses a severe threat to human life. Therefore, in order to ensure food safety and human health, it is of great practical importance to establish efficient methods for detecting antibiotics in the environment or biological samples [14].

### 1.2. Rapid Antibiotic-Detection Methods

Antibiotic resistance is mainly caused by the misuse, abuse, and overuse of antibiotics across various sectors, including poultry, agriculture, livestock, healthcare, industry, and environmental management. This poses a serious threat to human, animal, and ecological health. Therefore, antibiotics come from different sources, including hospitals [15], livestock [16], aquaculture [17], agriculture [18], wastewater [18], and more.

The liquid chromatography mass spectrometry/mass spectrometry (LC-MS/MS) method for the detection of penicillin-group antibiotic residues in food of animal origin (GB/T 21315-2007) was promulgated and implemented by the China Academy of Inspection and Quarantine Science (CAIQS) in 2008, and is a standard detection method in laboratories. Other methods include LC-MS/MS [19,20], high-performance liquid chromatography (HPLC) [21], ultraviolet liquid chromatography (HPLC-UV) [22], field-effect transistor (FET) [23,24], surface-enhanced Raman spectroscopy (SERS) [25], enzyme-linked immunosorbent assay (ELISA) [26], etc. Current state-of-the-art antibiotic-monitoring methods are achieved by optical/electrochemical/colorimetric/biological sensors with driver modules such as SERS, metal–organic frameworks (MOFs), localized surface plasmon resonance (LSPR), CRISPR/Cas, and fluorescent materials [27,28]. The physicochemical properties of these sensors can be adapted by optimizing the sensor platform design scheme, spiking concentration, and synthesis parameters with relatively short response time, ease of use, portability, and adequate sensitivity and accuracy. The detection efficiency of these sensors can be further improved by integrating modern information technologies such as artificial intelligence (AI) [29]. Used as a monitoring tool, they can effectively prevent the overuse of antibiotics in the agricultural sector and production sectors such as poultry farms.

In recent studies, optical and electrochemical sensing platforms have also been coupled with aptamers (Apt) to design aptasensors [30,31] that use ribonucleic acid (RNA) or deoxyribonucleic acid (DNA) aptamers for specific binding of targeted antibiotics [32,33]. Aptamer sensors are expected to detect specific antibiotics in a wide range of matrices with minimal sample pretreatment, have the advantages of rapidity, sensitivity, adjustability, versatility, and low cost, and provide miniaturized and portable sensing platforms [31], which overcomes the problems of traditional detection platforms such as large time consumption and costly, expensive, and complicated instrumentation, and low detection sensitivity and specificity; they are expected to promote the further antibiotic detection sensor. Table 1 summarizes the different methods used to detect antibiotics and compares the sensitivity of the different detection methods.

## 2. CRISPR/Cas Detection System

### 2.1. Introduction to the CRISPR/Cas System

The CRISPR/Cas are adaptive immune defense mechanisms in bacteria and archaea. The CRISPR/Cas system can be classified into Class-I and Class-II based on the types of effector proteins, which contain six isoforms: Type I, Type II, Type III, Type IV, Type V, and Type VI isoforms [45,46]. The Cas effector proteins of Class-I are multi-subunit proteins composed of complexes, including Type I, Type III, and Type IV isoforms. The Cas effector proteins of Class-II are composed of single subunits, including the Cas9 protein in Type II, the Cas12 protein in Type V, and the Cas13 protein in Type VI [47,48,49]. The Cas effector proteins of Class-II have a single structure that can efficiently achieve target recognition and cleavage, making them widely used for genome editing [50,51]. In these systems, the recognition of different target nucleic acids can be easily achieved by altering the crRNA sequence.

The first gene-editing tool to be developed was the Cas9 protein. In 2020, the Nobel Prize in Chemistry was awarded to French scientist Emmanuelle Charpentier and American scientist Jennifer A. Doudna for their discovery of one of the biggest tools in gene-editing technology, CRISPR/Cas9. Since then, research on the CRISPR/Cas9 system has been in full swing.

The CRISPR/Cas9 system consists of the Cas9 protein and guide RNA (gRNA). The Cas9 protein contains two major nuclease domains—the RuvC domain, which cuts noncomplementary DNA strands, and the HNH domain, which cuts complementary DNA strands; the gRNA serves a scaffolding function. The gRNA is a chimeric RNA formed by the combination of transactivated CRISPR RNA (tracrRNA), which functions as a scaffold, and specific crRNA, used to direct Cas9 to its target dsDNA. In this process, 5′-NGG-3′, the protospacer adjacent motif (PAM) sequences must be contained upstream of the targeting sequences [52]. Since PAM sequences are frequently found in most DNA sequences, Cas9 can target almost any gene with high specificity.

Currently, CRISPR/Cas-based nucleic acid assays are classified into two types in terms of principle: the first utilizes the property of Cas proteins such as Cas9 to recognize and bind dsDNA with high specificity, and the second utilizes the property of Cas proteins such as Cas13, Cas12, and other Cas proteins that activate trans-cleavage activity to cleave ssDNA or ssRNA nonspecifically after specifically recognizing the nucleic acid [53]. In 2016, Collins first introduced the CRISPR/Cas9 system to nucleic acid molecular diagnostics, developing a new technique to identify Zika virus lineages [54]. The method can provide precise genotypic information in hours and has attracted the attention of scientists. Researchers have developed a series of nucleic acid-detection protocols based on Cas9′s particular recognition and combined with the properties of dsDNA.

In 2018, Jennifer Doudna’s team published a related study on the CRISPR/Cas12 system in Science [55]. The CRISPR/Cas12 system consists of a single RuvC structural domain that mediates DNA cleavage at the far end of the PAM, labeled with the Cas12 gene, and some of them are mediated by single crRNAs such as CRISPR/Cas12a; some are co-mediated by crRNA and tracrRNA (or fused sgRNA alone), such as CRISPR/Cas12b [56,57]. As many as 11 isoforms (Cas12a-k) have been identified for the labeling genes of the CRISPR/Cas12 system, with recently discovered CasX classified as Cas12e, CasY classified as Cas12d, Cas14 categorized as Cas12f [58]; the most widely studied of these are Cas12a, Cas12b, and Cas12f [55,56,57,58,59].

Cas12a, also known as Cpf1, is a class of nucleic acid endonucleases mediated by a single crRNA that recognizes explicitly and shears dsDNA targets with PAM (5-TTTN-3′ or 5′-TTN-3), causing dsDNA targets to break and generate sticky ends. The recognition and shearing of ssDNA targets can be independent of PAM sequences. PAM sites are rich in thymines under the guidance of crRNA, activating CRISPR/Cas12a for targeted dsDNA cleavage (cis-cleavage) and activating CRISPR/Cas12a for non-targeted cleavage (trans-cleavage) [60]. To date, CRISPR/Cas12a-based tools have been widely used in many fields. Studies have shown that CRISPR/Cas12a has highly efficient trans-cleavage activity on ssDNA [61], a unique property that has been widely used in nucleic acid detection and has contributed to the improvement of CRISPR/Cas-based diagnostics (CRISPR-Dx) platforms by providing a strategy to improve specificity, sensitivity, and reaction speed [62].

The research related to CRISPR/Cas13 originated from a report published by Feng Zhang in Molecular Cell in 2015 [63], and his subsequent related report further elucidated that Cas13 has the function of cutting RNA, which formally unveiled the research and application of the CRISPR/Cas13 system.

The Cas13 protein does not require trans-activating RNA (tracrRNA) [49]. It has two distinct RNase activities: preprocessing of pre-crRNA into mature crRNA and shearing of the target RNA [64,65,66]. Cas13 is guided by a single crRNA, which catalyzes the maturation of pre-crRNA into mature crRNA, which recognizes specific pre-spacer sequence flanking sites (PFS, equivalent to the PAM sequence of DNA recognized by Cas9) in the non-target strand when the crRNA-Cas13a complex is formed. When the Cas13-crRNA complex recognizes PFS, the crRNA directs it to activate the CRISPR/Cas13 system by base complementary pairing with the target RNA. The target RNA is specifically sheared in cis, and nearby RNAs are trans-cleavage in a non-specific manner. At the time, this could be a promising tool for the detection of single-stranded RNA (ssRNA) virus detection [64,67].

Doudna’s group studied the Cas systems available in nature (Cas9, Cas12, and Cas13). They searched for uncharacterized Cas genes by creating a macrogenomic database of bacterial genomes, which led to the discovery of the Cas14 protein [68]. The Cas14 protein has been reported to target ssDNA and cleave ssDNA without PAM. The cas14 protein recognizes ssDNA, mediates the interaction of the target sequence with the target ssDNA, and cleaves ssDNA but not dsDNA or ssRNA. Similar to Cas9, the Cas14 protein requires tracrRNA and crRNA to target ssDNA [68]. Cas14 proteins are more specific in their cleavage efficiency than Cas9, Cas12, and Cas13 proteins that do not have a PAM region [58]. Therefore, this system meets all the criteria for high-fidelity genome editing.

### 2.2. CRISPR/Cas System for Detection of Nucleic Acid Targets

Because of its remarkable specificity and programmability in identifying and cutting particular DNA sequences, the CRISPR/Cas system has completely transformed the area of gene editing. Numerous studies have shown that the CRISPR/Cas system includes DNase/RNase characteristics in addition to its gene-editing ability, which makes it ideal for usage in the detection sector [69,70]. The nucleic acid signals of the target to be tested can be accurately recognized and targeted by gRNA/crRNA and Cas proteins. After recognizing ssDNA, dsDNA, or ssRNA targets, Cas12a/Cas13a/Cas14a proteins can exhibit nonspecific “trans-cleavage activity” towards nucleic acid sequences in the surrounding environment [61,71]. Numerous CRISPR/Cas-based nucleic acid-detection platforms, such as DNA endonuclease-targeted CRISPR trans reporter (DETECTR) [51], specific high-sensitivity enzymatic reporter unlocking (SHERLOCK) [72], one-hour low-cost multipurpose highly efficient system (HOLMES) [73], CRISPR/Cas-only amplification network (CONAN) [74], and others, have been successfully developed on the basis of these theoretical underpinnings.

The effector proteins of CRISPR/Cas12a (Cpf1) are RNA-directed enzymes that cleave both dsDNA and ssDNA. Similar to Cas9, Cas12a has been used for genome editing due to its ability to cleave targeted dsDNA. However, Cas12 (especially LbCas12a) stimulates nonspecific trans-cleavage activity by binding to its target DNA, which can completely degrade ssDNA molecules in the system. This target-activated transcutting activity is suitable for the molecular diagnosis of DNA and has contributed to the development of DETECTR methods [51]. For example, the trans-cutting activity of Cas12 was used as a diagnostic platform to diagnose Mycobacterium tuberculosis in high-sensitivity clinical samples [51]. Xu et al. also identified a DETECTR assay platform based on the recombinase polymerase amplification (RPA)-based DETECTR assay platform [75]. This method is rapid (<40 min), easy to implement, and accurate for identifying B. anthracis nucleic acids with a sensitivity close to 2 copies. Recent studies have also developed the DETECTR assay platform based on LtCas12a for precise and rapid detection of the human papillomavirus (HPV) 16/18 gene. Unlike AsCas12a and LbCas12a, which exert their cleavage activity by recognizing TTTV-containing PAM sites, limiting targeting, this LtCas12a utilizes unique TTNA PAM sites with equivalent cleavage capacity and specificity, thus providing new research directions for the development of new therapeutics and diagnostics.

Researchers also continue to improve the CRISPR/Cas system’s ability to detect nucleic acids at the molecular level rapidly. For example, HOLMES was developed to detect target DNA and RNA rapidly. Interestingly, HOLMES was further improved (HOLMESv2.0) to not only perform simple nucleic acid detection but also specifically differentiate between single nucleotide polymorphisms (SNPs), conveniently quantify target nucleic acids by a one-step system coupled with loop-mediated isothermal amplification (LAMP) at constant temperature, and accurately quantify target DNA methylation by a combination of Cas12b detection and bisulfite treatment [56,76,77]. However, the method relies on pre-amplification, which limits its development.

SHERLOCK, a Cas13a-based nucleic acid-detection platform, was developed to detect target RNA rapidly. In the SHERLOCK platform, a quenched fluorophore is added to the substrate, which is released and, therefore, fluoresces once the substrate cleaves, allowing target RNA detection [64]. Current CRISPR diagnostics typically combine isothermal amplification with CRISPR/Cas-mediated signal amplification and detection via RPA or cyclic LAMP. This combination dramatically improves diagnostic specificity and sensitivity.

To speed up detection, the researchers introduced a pathogen sample-processing method, heating an unextracted diagnostic sample to eliminate nuclease (HUDSON), that allows detection directly from body fluids without nucleic acid extraction. On this basis, SHERLOCK can detect Zika virus (ZIKV) and Dengue virus (DENV) directly from patient samples in 2 h without instrumentation, with detection limits as low as 1 copy/ul [78]. Subsequent research has shown that SHERLOCKv2 may leverage the selectivity of several Cas proteins, such as Cas12 and Cas13, to perform multiplexed nucleic acid detection of at least four distinct targets in a single process [79].

Pena recently reported two novel Cas12 enzymes, SLK9 and SLK5-2 [80], which exhibit high activity at 60 °C. Real-time nucleic acid-detection methods (real-time SLK) can be achieved using LAMP for detection. This method can simultaneously detect SARS-CoV-2 and human-based controls, with a detection limit of 5 copies/mL and a detection time of 30 min [81].

Impressive advances in the field have led scientists to utilize CRISPR/Cas technology creatively as a susceptible diagnostic platform. Quan et al. developed FLASH (Finding Low Abundance Sequences by Hybridization) by exploiting the efficiency, specificity, and programmability of Cas9 [82]. The target sequence is enhanced in the background and utilized for further sequencing using targeted amplification.

CRISPR/Cas-based methods have achieved unprecedented precision in gene editing, and it is important to consider the risks of their potential misuse [83,84]. This is because such misuse may raise ethical and social issues. Sound regulations can prevent misuse, but overly strict measures may hinder the development of CRISPR/Cas technology. Therefore, it is important to strike a balance between avoiding misuse and promoting scientific progress [85].

### 2.3. CRISPR/Cas System for Non-Nucleic Acid Target Detection

The CRISPR/Cas system has revolutionized gene editing. However, ions, cells, proteins, and small molecules are also biomarkers closely related to life and health. Traditional detection methods are time-consuming, costly, and have low sensitivity and specificity. In contrast, CRISPR/Cas-based sensors can provide inexpensive, rapid, and accurate detection solutions. Introducing this technology not only expands the application field of CRISPR gene-editing technology but also provides a new idea for the susceptible and specific detection of non-nucleic acid targets.

**Figure 1:**
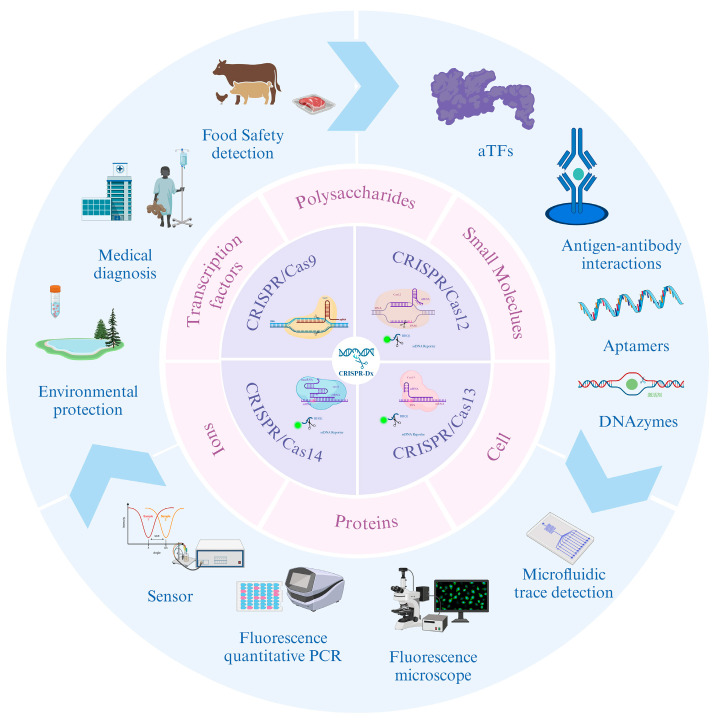
Non-nucleic acid target-detection strategy-based on CRISPR-Dx. The innermost circle describes the principle of action of the four CRISPR effector proteins; the middle circle represents the non-nucleic acid targets detectable by the CRISPR effector proteins; and the outermost circle describes the non-nucleic acid targets in the environment that are converted by the bio-transduction element into recognizable nucleic acid signals, which are then outputted on the constructed CRISPR detection platform. Created in https://BioRender.com.

Non-nucleic acid target-detection strategies based on the CRISPR-Dx system typically utilize the trans-cutting activity of CRISPR/Cas proteins for detection. During the detection process, non-nucleic acid targets must be converted into recognizable nucleic acid signals by biotransduction elements that can be taken up by CRISPR/Cas [86]. The use of nucleic acid sequences to activate CRISPR/Cas cleavage of target DNA, along with activation of CRISPR/Cas trans-cleavage activity to produce detectable output signals [87], enables the detection of non-nucleic acid targets (Figure 1).

In 2019, Liang et al. published a small molecule-detection platform named CaT-SMelor, CRISPR/Cas12a and small molecule detector mediated by bacterial ectopic transcription factor (aTF) [88]. By combining the ssDNA cleavage ability of CRISPR/Cas12a and the competitive binding activity of aTFs on small molecules and dsDNA, this high-throughput small-molecule-detection platform can detect small molecules, including uric acid and p-hydroxybenzoic acid and their structurally similar analogs, with a limit of detection down to the nanomolar level.

In 2023, Yee et al. introduced a CRISPR/Cas-based aptasensor for the susceptible and specific detection of the antibiotic agent ampicillin [41]. They developed the aptamer sensor using the CRISPR/Cas system using three different ampicillin-specific aptamers. An ssDNA activator designed according to the aptamers binds to the aptamers by complementary base pairing, and the CRISPR/Cas system is activated by the release of bound ssDNA after the aptamers are attracted to the ampicillin target during detection. It activates the trans-cleavage activity of Cas12a, cleaves the probe, and outputs a fluorescent signal. The detection limit reaches the picomolar level, and the detection time is less than 30 min.

In addition, Wu et al. [89] investigated a new strategy called Nazyme-Activated CRISPR/Cas12a with Circular CRISPR RNA (NA3C), which utilizes cyclic topology of crRNAs to “lock” the CRISPR/Cas12a cis-cleavage and trans-cleavage activity and activates CRISPR/Cas12a cleavage by linearizing the cyclic current in a reaction system using a nuclease with RNA cleavage activity. The use of NA3C for the detection of E. coli in the urine of clinical patients eliminates the need for a culture step. It provides a diagnostic sensitivity of up to 100% and a specificity of 90%.

In addition to metabolites and antibiotics, CRISPR-Dx has also been developed to detect ions, polysaccharides, proteins, cells, transcription factors, etc. (Figure 1) [86]. CRISPR-Dx has the advantages of high specificity, high sensitivity, and programmability; however, unlike nucleic acid targets, non-nucleic acid target detection is currently in the early stages of development, and it still faces many challenges.

## 3. Application of CRISPR Technology for Antibiotic Detection

### 3.1. CRISPR/Cas for the Detection of Antibiotic-Resistance Genes

The misuse and improper disposal of antibiotics accelerate the formation of mutant and multidrug-resistant bacteria (MDR) (also known as “superbugs”) [90,91]. The mechanism of antibiotic resistance in bacteria can be attributed to horizontal transfer and vertical transmission of resistance genes on mobile genetic elements (MGEs), such as plasmids, between bacteria [92,93]. Normal bacteria can develop antibiotic resistance through changes in their chromosomes or genetic material [94]. Serious infections and molecular alterations in the host genome may result from this [95]. Effective infection prevention relies on the early identification of antibiotic-resistant bacteria, and rapid detection of specific drug-resistant genes using straightforward techniques is crucial for promptly addressing antibiotic resistance.

Traditional culture methods are the gold standard for bacterial detection, including bacterial culture and plate-counting processes for standard drug sensitivity testing [96]. However, these assays are complex, time-consuming, labor-intensive, and have relatively low sensitivity. With the discovery of an increasing number of antibiotic-resistance genes in both Gram-negative and Gram-positive bacteria, gene-based assays have emerged [97,98]. Commonly used assays are primarily based on polymerase chain reaction (PCR) [99]. Although sensitive and fast, PCR is prone to nonspecific amplification during thermal cycling, leading to false positive results and low specificity. Therefore, there is an urgent need to develop a method that is simple, rapid, and specific for detecting antibiotic-resistance genes.

#### 3.1.1. Cas9 for the Detection of Drug-Resistance Genes

The use of CRISPR/Cas for gene editing has undergone exponential growth over the past few years. It has been rapidly used for genome editing in a variety of various cell types and experimental platforms [100], with some studies suggesting a greater potential for targeting ARGs. The rapid increase in antibiotic-resistant bacteria poses a significant threat to human health. It has the potential to undermine most of the gains of modern medicine in the near future [101]. Rapid and sensitive diagnosis of resistance can help healthcare professionals administer appropriate treatment directly, use existing antibiotics more effectively, and avoid the use of “last resort” antibiotics [102]. Therefore, a method for rapid and accurate detection of drug-resistance genes is important for the development of modern medicine.

Since the characterization of the Cas9 protein from the Streptococcus pyogenes in 2012 [103], the exquisite and programmable specificity of the CRISPR system has inspired many new uses of its enzyme beyond genome engineering. There are also more applications in antibiotic detection, which is in full swing in detecting drug-resistant genes and their use in detecting bacterial pathogens (Table 2).

**Figure 2:**
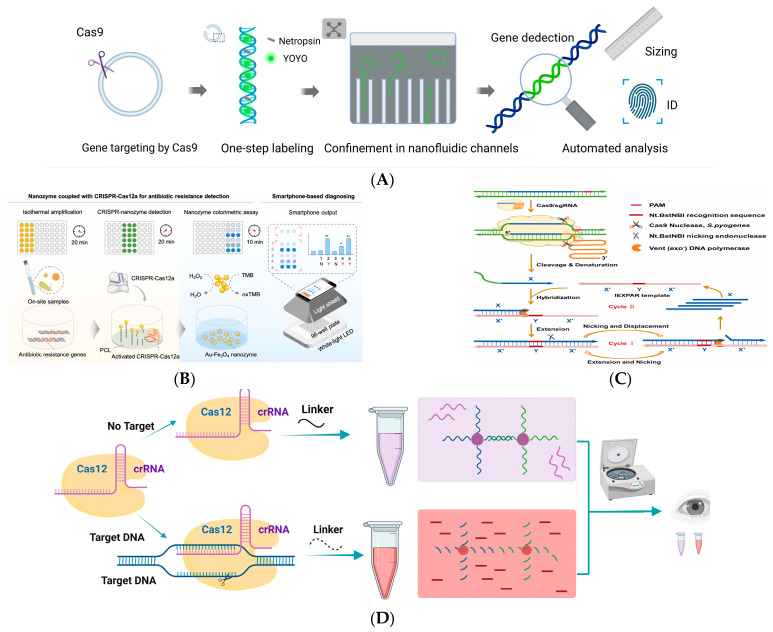
The CRISPR/Cas system-based detection platform for antibiotic-resistance genes. (**A**) Specific DNA was cut using the Cas9 enzyme, the fluorescent dye was added, and then the plasmid was stretched in the nano-channel, and the information was observed by fluorescence microscope. Copyright Vilhelm Müller. (**B**) The resistance gene fragment was amplified by RPA technology to activate the CRISPR/Cas12a system, followed by colorimetric detection using Au-Fe_3_O_4_ nanoenzymes.(* *p* < 0.05, ** *p* < 0.01) Reproduced with permission from Ref. [108]. Copyright 2023 Elsevier. (**C**) CRISPR/Cas9 recognizes and cleaves drug-resistant genes and activates IEXPAR amplification for rapid detection. Reproduced with permission from Ref. [106]. Copyright 2022 Elsevier. (**D**) The presence of target ARGs activates CRISPR/Cas12a to degrade the crosslinker, making the solution red. Without target genes, it turns purple due to the undegraded crosslinker. Reproduced with permission from Ref. [110]. Copyright 2023 American Chemical Society.

Müller et al. introduced a method for optical DNA mapping using a single plasmid within a nanofluidic channel [104]. This approach uses the cleavage capability of Cas9 to convert the circular plasmid into a linear form to identify resistance genes (Figure 2A). CRISPR/Cas9 is equipped with a gRNA to improve gene identification, which can be linearized at a particular location on the circular plasmid and subsequently identified by optical DNA mapping. In the research process, the researchers discussed optimizing this detection method for future clinical applications by combining multiple gRNAs that target different genes associated with resistance to the same class of antibiotics.

The assay is suitable for low sample concentrations, and can reveal as much information as possible in a single experiment. At the same time, the assay can measure the size of each plasmid, provide a fingerprint that can be used to identify and track the plasmid and determine the presence or absence of the resistance gene of interest on the plasmid. However, the comprehensive information obtained would require several different techniques and take up to a week to complete, so the method needs to be further developed for future applications.

Using the principles of this technique, Nyblom et al. identified Escherichia coli and Klebsiella pneumoniae directly from patient samples at the strain level [105]. They identified strains or subtypes simultaneously and characterized the corresponding plasmids by restricting antibiotic-resistance genes from the targeted plasmids with Cas9. This optical DNA-profiling assay can rapidly provide comprehensive diagnostic information to optimize early antibiotic treatment regimens and open the way for future precision medicine management.

In 2019, Quan et al. developed a CRISPR/Cas9-based detection technology called FLASH. Due to the high sensitivity and specificity of Cas9, this technology can identify antimicrobial resistance genes in various clinical samples, with a detection limit of 1.9aM [82]. FLASH can reveal the sequence identity of the target DNA, which is an advantage over other detection platforms.

In 2023, Qin et al. developed a novel technique for identifying antibiotic-resistance genes using the CRISPR/Cas9-induced isothermal exponential amplification reaction (IEXPAR) [106]. In the reaction system, antibiotic-resistance genes can be identified by CRISPR/Cas9 and cleaved into two short fragments with free 3ʹ-OH end. The utilization of a cleaved DNA template initiates efficient exponential amplification of IEXPAR, which directly identifies antibiotic-resistance genes under isothermal conditions (Figure 2C). This detection is rapid and precise. As for antibiotic-sensitive bacteria, due to the absence of antibiotic-resistant genes, the CRISPR/Cas9 system cannot cleave any DNA, and no subsequent amplification reaction occurs, ensuring the high specificity of the method. In the experiment, after about 30 min of amplification, antibiotic-resistant genes as low as 100 fM could be sensitively detected, with a detection limit of 81 fM. The method was also applied to the detection of antibiotic-resistant bacteria in actual biological samples. Antibiotic-resistant and antibiotic-sensitive bacteria could be identified under isothermal conditions, and the operation was simple.

#### 3.1.2. Cas12 for the Detection of Drug-Resistance Genes

CRISPR/Cas12a is widely used for genome editing due to its ability to perform precise double-stranded DNA cleavage. Curti et al. constructed a CRISPR/Cas12a-based assay platform [107] in which DNA target sequences correspond to carbapenemase-resistance genes. The system detected each target on a picomolar scale in 10 min. Furthermore, considering that the background of genomic DNA (gDNA) and low levels of free targets in blood can lead to inhibition of reaction, the researchers’ strategy of combining RPA and CRISPR/Cas12 allowed the detection of carbapenem-resistance genes, specifically Klebsiella pneumoniae carbapenemase (KPC), New Delhi metallo β-lactamase (NDM), in less than an hour. The sensitivity and accuracy were comparable to that of RT- qPCR. Validation was carried out using portable test strips with a 100% correlation between the fluorescence test and the portable test strips.

The development of superbugs, a serious threat to the health of all living things, may be accelerated by the accumulation and dissemination of ARB in the environment. Chen et al. created a colorimetric method for identifying ARGs by combining the Au-Fe_3_O_4_ nanozyme with the CRISPR/Cas12a nucleic acid-specific recognition capability [108]. The trans-cleavage activity of the CRISPR/Cas12a system in the research system is triggered by the recognition of the target resistance gene. This results in the release of the Au-Fe3O4 nanozyme, the oxidation of 3,3,5,5-tetramethylbenzidine (TMB), and a change in the color of the solution from transparent to blue (Figure 2B). Diagnostic signals can be recorded and analyzed using a smartphone and the signal recognition terminal. Chloramphenicol, ampicillin, and kanamycin-resistance genes can be found using this technique. Due to its high sensitivity (less than 0.1 CFU/μL) and speed (less than 1 h), this detection technique enables the quick and precise identification of ARB or ARG in the field, enabling flexible and efficient monitoring and management of antibiotic contamination [109].

In 2023, Kasputis et al. also developed a CRISPR/Cas12a-based assay [110] to detect ARGs in wash water collected from food-processing plants. DNA-functionalized AuNPs were crosslinked during the assay with ssDNA cross-linker. Degradation of the cross-linker using Cas12a trans-cleavage activity alters the optical properties of AuNPs to produce a simple visual readout (Figure 2D). This assay can still efficiently detect three representative ARGs without DNA amplification with a detection limit of 5 nM or less.

Mao et al. combined CRISPR/Cas12a and LAMP to construct a portable biosensor [111]. For this purpose, primer sets and detection systems were also specially designed, and finally, the output signals were analyzed using fluorescence and lateral flow. Typical ARG ermB was used as the target in the study, and the detection limit was as low as 2.75 × 10^3^ copies/μL. This simple-to-operate and low-cost biosensor is of significant use for large urban areas with many wastewater-treatment plants and rural regions with limited resources. It also provides a new treatment method for detecting ARGs in wastewater.

ARB pose a significant threat to global health, with bacteria producing NDM being particularly problematic because they are resistant to most beta-lactam antibiotics. In this regard, Shin et al. proposed a fluorescent assay based on PCR-coupled CRISPR/Cas12a that can detect NDM genes (bla_NDM_) produced in bacteria [112]. Thanks to its self-designed gRNA, this CRISPR/Cas12a system can efficiently cleave both the PCR amplification product and the fluorescent probe simultaneously, thus generating a fluorescent signal. The detection performance of this method is excellent, with a detection limit of 2.7 CFU/mL, which is 100 times higher than that of the conventional gel electrophoresis PCR method. Furthermore, this assay detects ARGs in food samples and performs better than previously published quantitative fluorescent quantitative PCR assays.

#### 3.1.3. Cas13 and Others

In recent years, the CRISPR/Cas system has been rapidly developed for pathogen nucleic acid detection. In 2017, Zhang’s team established the SHERLOCK molecular diagnostic platform for the first time by combining the CRISPR/Cas13a system with isothermal amplification technology [113]. On the basis of this, researchers have established a series of highly sensitive and specific nucleic acid-detection methods for various viruses and bacteria by combining different amplification and visualization detection techniques.

In response to the fact that current drug-resistance gene detection usually relies on specialized testing facilities and equipment, rapid detection technology for drug-resistance genes still needs to be developed. Qiang Hu et al. developed a CRISPR/Cas13-based rapid detection technique for the rapid identification of the mecA-resistance gene in Staphylococcus aureus [114]. The researchers developed the recombinase-aided amplification (RAA) primers and crRNA, subsequently employing the easy read-out and sensitive enhanced (ERASE) technology to identify the mecA gene. The method demonstrates a minimum detection limit of 10 copies/μL and achieves 100% concordance with the results of drug susceptibility tests and qPCR detection. The detection platform established by Yan et al. using the same method, named SHIELD (easy-read H. pylori easy-read dual detection) [115], was used to detect the clarithromycin-resistance gene. The detection limit can reach 50 copies/μL.

Both of the above are based on CRISPR technology and, simultaneously, combined with ERASE nucleic acid-detection test strips, reducing dependence on specialized testing instruments and equipment and allowing for more convenient and quicker detection of bacterial drug-resistance genes. It has excellent development prospects to prevent the spread of ARB and to monitor the progress of bacterial drug resistance in real-time. However, this method is the same as most current bacterial nucleic acid-detection methods, which require the extraction of nucleic acids from bacterial samples prior to detection, and the current rapid extraction of bacterial nucleic acids usually relies on equipment such as centrifuges and magnetic racks or requires the purchase of the corresponding nucleic acid extraction kits. This strategy not only increases the complexity of the testing process but also increases the cost of testing. In contrast, Ortiz-Cartagena et al. used a new assay based on LAMP & CRISPR/Cas13a for the detection of carbapenem-resistant genes in clinical samples [116], which has high specificity and sensitivity, can be performed without the need for specialized methods for RNA extraction, and shows 100% accuracy for the detection of drug-resistant genes. Furthermore, it does not require specific equipment or trained personnel, and the sample per reaction is small, making it convenient and inexpensive. Therefore, this CRISPR/Cas-based assay kit will be highly competitive in the commercial market in the future.

Due to the widespread use of antibiotics, bacteria have developed various defense methods, such as restriction-modification systems and CRISPR/Cas mechanisms. As the innate and adaptive immune systems of bacteria, these two mechanisms have a significant impact on the spread of ARGs. They are considered inhibitors of horizontal gene transfer in bacteria [117,118,119]. Pathogens with the CRISPR/Cas system have been found to be less likely to carry ARGs than pathogens lacking this defense system [120], a finding that provides new strategies for combating the spread of ABR.

**Table 2:** CRISPR/Cas systems for the detection of drug-resistance genes.

Cas Effectors	Detection Platform	Whether to Amplify or Not ^1^	Target	LOD	Time (min)	Ref.
CRISPR/Cas9	CRISPR/Cas9 combined with optical DNA mapping	N	ESBL gene family bla_CTX-M_; The carbapenemase gene families bla_NDM_ and bla_KPC_	NR	NR	[104,110]
	FLASH	Y	antimicrobial-resistance genes	1.9 aM	NR	[82]
	IEXPAR	Y	mecA gene in real genomic DNA samples	81 fM	NR	[106]
CRISPR/dCas9	CRISPR/dCas9-SERS	N	macrolide antibiotic-resistant macB gene in milk	11.9 fM	NR	[121]
CRISPR/Cas12a	RPA amplification	Y	carbapenemases-resistance genes such as KPC, NDM and OXA	100 aM	<120	[107]
	Au-Fe3O4 nanozyme coupled with CRISPR/Cas12a	N	Kana-resistance genes; AMPI-resistance genes; Chloramphenicol-resistance genes	<0.1 CFU/μL	<60	[108]
	Colorimetric detection based on CRISPR/Cas system	N	ermB; sul1; tetW	5 nM	50	[110]
	Portable biosensor combining CRISPR/Cas12a and LAMP	Y	ermB in wastewater	2.75 × 10^3^ copies/μL	70	[111]
	RPA coupled with CRISPR/Cas12a platform	Y	Colistin Resistance Gene mcr-1	1.6 × 10^3^ CFU/mL	60	[122]
	Cas12a/3D DNAzyme colorimetric paper sensor	Y	NDM-1 gene encoding metallo-β-lactamase	100 fM	<90	[123]
	Cas12a dual detection platform (Cas12a-Ddp)	Y (PCR&RPA)	mcr-1 and invA genes	33/214 fM	45/75	[124]
	CRISPR/Cas12a cou- pled with PCR	Y	bla_NDM_ in Carbapenem-Resistant Enterobacterales	2.7 CFU/mL	NR	[112]
CRISPR/Cas13a	CRISPR/Cas system Combining ERASE	Y	Staphylococcus aureus mecA-resistance gene	10 copies/μL	NR	[114]
	RAA-CRISPR/Cas13a Fluorescence Detection System	Y	A2142G and A2143G mutant DNAs causing clarithromycin resistance	50 copies/μL	NR	[115]
	Combines RPA and CRISPR/Cas13a: one-tube and two-step reaction	Y	mexX gene in P. aeruginosa	10 aM/1 aM	5/40	[125]
	RPA-Cas13a assay	Y	bla_KPC_	2.5 copies/μL	60	[126]
	LAMP-CRISPR/Cas13a-based assay	Y	OXA-48 and GES Carbapenemases	NR	<120	[116]

^1^ In the third column, Y = Amplify; N = Not Amplify.

### 3.2. CRISPR/Cas for the Detection of Antibiotic Molecules

*Nature* has recognized antiretroviral enzymes based on CRISPR/Cas as one of the top seven technologies to watch in 2022 [127]. CRISPR/Cas-based biosensing technology is indeed promising, but currently, it is mainly limited to detecting nucleic acid targets. The development of detection systems for non-nucleic acid targets is still in its infancy, especially in detecting antibiotic molecules with fewer applications.

**Figure 3:**
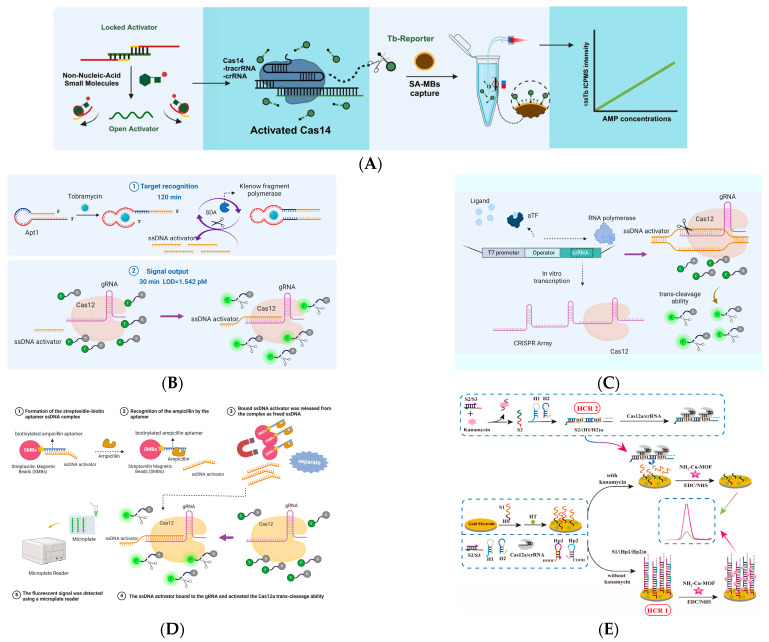
CRISPR/Cas-based detection platform for antibiotics. (**A**) The AMP aptamer releases an activator upon target binding, activating Cas14 to cleave a probe, and AMP concentration is quantified by ICPMS detection of terbium isotope intensity. Used with permission of © The Royal Society of Chemistry 2021, from [128]. Permission conveyed through Copyright Clearance Center. (**B**) Tobramycin-bound aptamer triggers SDA to generate ssDNA activators that activate CRISPR/Cas12a to cleave reporter probes and output signals. Reproduced with permission from Ref. [42]. Copyright 2021 Elsevier. (**C**) In the presence of the ligand, dissociation of the aTF allows transcription of the CRISPR array, activating CRISPR/Cas12a and cleaving the probe, outputting a signal. Copyright©2022 Ahmed Mahas. Published by American Chemical Society. This publication is licensed under CC-BY 4.0. (**D**) The aptamer recognizes ampicillin and releases the ssDNA activator, which activates the trans-cleavage of Cas12a and outputs a fluorescent signal. Reproduced with permission from Ref. [41]. Copyright 2023 Elsevier. (**E**) Without kanamycin, S1 triggers HCR1, generating a strong electrical signal. In the presence of kanamycin, activation of CRISPR/Cas12a blocks HCR1 and reduces the electrical signal. Reproduced with permission from Ref. [132]. Copyright 2024 Elsevier.

An elemental probe-based CRISPR/Cas14 detection platform (Figure 3A) for non-nucleic acid targets was first proposed by Hu et al. in 2021 [128]. Metal isotope detection was combined with CRISPR/Cas14 biosensors to detect the non-nucleic acid target ampicillin sensitively. In this study, a fluorescence-quenching pair-labeled probe (FQ) was designed and optimized, and the target nucleic acid could be quantified using the fluorescence signal of the incidentally cleaved FQ fragment. This method quantifies trace ampicillin in aqueous solution in 45 min at room temperature (25 °C) with a detection limit as low as 2.06 nM, which is excellent in anti-interference testing and complex matrix detection.

At the same time, Lv et al. published a metal-labeled CRISPR/Cas12a bioassay, and applied it to an ultra-sensitive and highly selective assessment of antibiotic bioaccumulation in wild fish [43]. The methodology integrates an elementally labeled CRISPR/Cas12a reporter probe with incidental cleavage activity. The target kanamycin was recognized and captured by a “lock activate” system, followed by the release of an activator chain to activate the incidental cleavage activity of Cas12a, which cleaves the free metal reporter (Tm-Rep). Uncleaved probes and cleaved biotin-modified molecules were captured using streptavidin-coated magnetic beads, and the remaining probes were available for quantitative detection of metal isotopes by inductively coupled plasma mass spectrometry (ICPMS). In this study, kanamycin was used as the detection target, and the detection limit was as low as 4.06 pM in 30 min.

Both methods mentioned above utilize the rare elements terbium (159Tb) and thulium (169Tm). However, both 159Tb and 169Tm are rare earth elements with minimal reserves and are war-prepared materials. This limitation could have been more conducive to large-scale replication of the method. The researchers then turned their attention to aptamers, coupling optical and electrochemical sensing platforms with aptamers [19,20] and using RNA or DNA aptamers for specific binding to targeted antibiotics [21] as a way to detect particular antibiotics. Based on this idea, in 2022, Li et al. constructed two ultrasensitive biosensors (sensor-ss and sensor-ds) based on the CRISPR/Cas system [42], which consisted of heterologous aptamer probes integrated with the CRISPR/Cas12a system for the detection of antibiotic tobramycin. Both sensors utilize hairpin DNA containing tobramycin aptamer sequences as target recognition probes and further generate signal-transduction sequences.

The two sensors vary in their target-recognition and signal-amplification methodologies. In sensor-ss (Figure 3B), ssDNA activates the trans-cutting activity of CRISPR/Cas12a, resulting in the cleavage of the FQ probe and an enhanced fluorescence signal. The dsDNA with the PAM sites at the sensor ds functions as a signal-transduction sequence, identified by CRISPR/Cas12a, resulting in the cleavage of the FQ probe. In the signal-amplification strategy, sensor-ss utilized a strand displacement amplification (SDA) reaction for signal augmentation, while sensor-ds did not incorporate a DNA-amplification method. By optimizing the DNA sequence and reaction parameters, the fluorescence response of sensor ds exhibited a linear correlation with the tobramycin concentration (10–300 pM), achieving a limit of detection (LOD) as low as 3.719 pM. Despite sensor ss exhibiting superior sensitivity (LOD = 1.542 pM) compared to sensor ds, rapid attainment of fluorescence signal saturation led to a limited linear response range (5–30 pM). This CRISPR/Cas12a-based assay is visually interpretable under UV light for on-site detection. The fabricated sensor-ds were utilized to identify tobramycin in milk and lake water samples. This target-recognition and signal-amplification technology offers a promising approach for developing a multipurpose sensing platform to detect other non-nucleic acid targets.

With the idea of developing a sensor for the CRISPR/Cas-based on aptamers, in the same year, Mahas et al. introduced an aTF into the CRISPR/Cas system to detect antibiotics [129]. They developed a simple, rapid, sensitive, and field-deployable small molecule-detection platform based on the CRISPR/Cas12a-aTF biosensor for tetracycline. In this biosensor, aTF binds to an operon sequence that hinders the in vitro transcription process. In the presence of tetracycline, the tetracycline binds to the aTF, causing the aTF to be released from the operator sequence. As a result, the in vitro transcription process can proceed normally, resulting in the acquisition of crRNA to activate the CRISPR/Cas12a system and generate fluorescent signals (Figure 3C). This assay platform is a valuable addition to the development of CRISPR/Cas-based, cell-free biosensors with great potential for detecting non-nucleic acid small molecules in situ.

In 2023, Yee et al. developed aptamer sensors (Figure 3D) for the susceptible and specific detection of the antibiotic agent ampicillin by the CRISPR/Cas system using three different ampicillin-specific aptamers [41]. The ssDNA activators bind to aptamers by complementary base pairing. The aptamer attracts the ampicillin target and releases bound ssDNA, activating the CRISPR/Cas system. It activates the trans-cleavage activity of Cas12a, cleaves the probe, and outputs a fluorescent signal. The method can be completed in 30 min with a detection limit of 0.01 nM. The sensor is also sensitive to ampicillin under complex substrates.

Chen et al. developed a portable biosensor detecting kanamycin based on glucometer and CRISPR/Cas12a [130]. With a detection limit of 1 pM, this technique for the on-site and low-cost monitoring of antibiotic residues in water samples using equipment readily available to the public is a significant discovery.

Researchers have recently combined hybridization chain reaction (HCR) with the CRISPR/Cas12a system to construct more flexible detection platforms. For example, Zhu et al. combined NH_2_-Co-MOF as electrocatalytic active material, HCR, catalytic hairpin assembly (CHA), and CRISPR/Cas 12, a technology to construct a sensitive and label-free electrochemical sensor for the detection of ampicillin [131], with a detection limit of 1.60 pM.

The detection platform constructed by Zhang et al. using the same method (Figure 3E) can reach a detection limit of 60 fM and 10 fM when used to detect the antibiotics kanamycin and ampicillin, respectively [132]. This electrochemical sensor, which uses HCR-triggered CRISPR/Cas12 with NH_2_-Cu-MOF as the signaling molecule, has considerable advantages. Initially, NH_2_-Cu-MOF, characterized by high electroactivity and accessible coupling properties, is used as an electrochemical signaling marker without the incorporation of additional redox mediators, thus increasing the output of the electrochemical signal and streamlining the structural complexity of the sensing system; subsequently, HCR was integrated with CRISPR/Cas12a to establish a cascade amplification circuit, which enhances the efficiency and sensitivity of the signal amplification of the sensing system. We summarized the reports published in recent years on the use of CRISPR/Cas for molecular detection of antibiotics (Table 3) and compared the sensitivity of the different detection methods.

## 4. Summary and Prospects

The emergence of the CRISPR/Cas system has revolutionized gene-editing technology and reshaped the landscape in the field of analysis and assays. The CRISPR/Cas system has rapidly evolved and is used to design powerful molecular diagnostic tools. CRISPR/Cas-based biosensors outperform traditional techniques in terms of accuracy, versatility, portability, timeliness, and efficiency due to specific target recognition and cis/trans cleavage activity. Therefore, by combining novel materials, novel methods, and novel sensing technologies, CRISPR/Cas biosensing systems are expected to satisfy a variety of needs and contribute to a number of domains, including environmental analysis, food safety testing, and disease detection.

However, the current technology platform based on the CRISPR/Cas system for antibiotic detection still faces several problems to be solved: (1) Most methods need to be combined with nucleic acid-amplification techniques (such as RPA, LAMP, etc.) to improve detection sensitivity. However, this process still needs to be performed step by step, which introduces the risk of contamination and dramatically increases detection time and workload. Although studies have been conducted to develop amplification-free nucleic acid-detection platforms, there are still problems, such as low detection limits. Integrated, fully enclosed microfluidic chips and devices that integrate isothermal amplification and signal output should be further explored to improve their clinical applications. (2) PAM sequence limitation. Most of the target sequences recognized by Cas12 are highly dependent on PAM sequences, limiting its detection’s versatility. In addition to introducing PAM sequences using amplification primers, more Cas proteins that are not strictly dependent on PAM sites should be identified to increase the flexibility of the assay. (3) False positive results. False positive results occur due to off-target cleavage by binding crRNA to nontarget sequences. The accuracy of the detection results can be ensured by the careful design of the crRNA to improve the target-cutting effect. (4) High-throughput multi-target detection. Most detection platforms based on the CRISPR/Cas system can only achieve qualitative detection of a single marker for a single sample, and it is challenging to realize multi-target quantitative detection for multiple samples. Based on the close correlation between the concentration of antibiotics and drug-resistant genes and the sensing strength of the CRISPR/Cas system-detection signals, the combination of microfluidics, microdroplets, microarrays, and other technologies with optical sensors and other ultrasensitive detection technologies to build a new type of detection platform will promote the solution of this problem. (5) The detection system should be standardized. The current CRISPR/Cas-based detection technology has not yet been formally standardized, and no approved products have been approved However, this situation will change with the growing maturity of this detection technology.

In summary, this paper points out the current development, challenges, and future directions of the CRISPR/Cas toolbox and its use in antibiotic detection for nucleic acid and non-nucleic acid target detection. The functionality and detection utility of the CRISPR/Cas system, especially its significant applications in antibiotic drug detection, are mainly introduced. A thorough overview of the CRISPR/Cas-based antibiotic-detection technique has not yet been published. This review provides a comprehensive overview of the development of this new technology in detecting nucleic acid and non-nucleic acid substances, focusing on its application in detecting antibiotic-resistance genes and antibiotic molecules. It provides a reference for controlling and reducing the expansion of antibiotic resistance.

## Figures and Tables

**Table 1:** Commonly methods used for antibiotics detection.

No.	Detection Methods	Readout Detector	Target	Detection Range	LOD	Real Sample	Time (min)	Ref.
1	National Standard Method -LC-MS/MS	MS	Methicillin	NR	0.1 μg/kg	Food of animal origin	NR	GB/T 21315-2007
2	LC-MS/MS	MS	Ampicillin(AMPI)/clarithromycin	4 × 10^5^~ 2.5 × 10^7^ nM	0.14~59.8 μM	Jawbone	NR	[19]
3	HPLC	Chromatograph	Sulfadiazine	50~500 ng/mL	22.4 ng/mL	Milk	NR	[21]
4	ELISA	Smartphone	Tetracycline chloromycetin	1~10^3^ ng/mL; 0.1~100 ng/ml	0.5 ng/mL 0.05 ng/mL	Milk/fish	NR	[34]
5	SERS	Raman spectrometer	loxacin	NR	15.8 μg/L	Aquatic products (fish)	30	[35]
6	SERS	SERS-ICA test strip	Sulfadimethoxine	0.1~ 10^3^ pg/mL	0.1 ng/L	Milk	15	[36]
7	FET	FET	Ampicillin	10^−12^~ 10^−6^ M	0.556 pM	Creek	3	[24]
8	Aptamer-modified graphene field-effect transistors (Apt-SGGT)	Digital seismograph	Tetracycline	NR	2.073 pM	Skim milk	8	[23]
9	MOF Fluorescence Sensor	Smartphone	Fluoroquinolone	0~90 μM	16 nM	NR	20	[37]
10	MOF Electrochemical Sensors	Electrochemical workstation	Ciprofloxacin	2.5~ 100 µM	3.29 nM	Tap water/seawater	NR	[38]
11	Long Afterglow Optical Sensors	Spectrophotometer	Kanamycin	1 pg/mL~ 5 ng/m L	0.32 pg/mL	Milk/honey/powdered milk	90	[39]
12	Long Afterglow Markless Sensors	Fluorescence spectrophotometer	Furacilinum	0.1~50 mM	5 nM	Milk/Dianchi water samples	NR	[40]
13	CRISPR/Cas12a Light Sensors	Microplate Reader	Ampicillin	0.01 nM~ 500 nM	10 pM	Milk/eggs/honey	30	[41]
14	CRISPR/Cas12a Biosensor	Spectrophotometer	Tobramycin	10~300 pM	3.719 pM	Milk/lake water	40	[42]
15	Metal-labeled CRISPR/Cas12a biosensors	ICPMS	Kanamycin	8~120 pm	4.06 pM	Wild fish	30	[43]
16	Microfluidic Sensors	UV-visible spectrophotometer	Kanamycin	0.8 pg/mL~10 ng/mL	0.3 pg/mL	Milk/fish	NR	[44]

**Table 3:** CRISPR/Cas for the detection of antibiotic micromolecules.

CAS Type	Detection Object	Sensing Method	Readout Detector	Detecting Linear Range	LOD	Time (min)	Real Sample	Ref.
CRISPR/Cas14	Ampicillin	Metal isotope labeling	ICPMS	NR	2.06 nM	45	NR	[43]
CRISPR/Cas12a	Kanamycin	Metal isotope labeling	ICPMS	8–120 pM	4.06 pM	30	Wild fish	[128]
	Tobramycin	Aptasensor	Spectrophotometer	10–300 pM	3.719 pM	40	Milk/lake Water	[42]
	Tetracycline	aTF	Smartphone	NR	2 μM	NR	NR	[129]
	Ampicillin	Aptasensor	Microplate reader	0.01–500 nM	10 pM	30	Milk/eggs/honey	[41]
	Kanamycin	Aptasensor	glucometer	1pM–100 nM	1 pM	NR	Water sample	[130]
	Ampicillin	HCR/ Electrochemical sensor	Electrochemical workstation	5 pM–100 nM	1.60 pM	160	Milk/ livestock wastewater	[131]
	KanamycinAmpicillin	HCR/ Electrochemical sensor	Electrochemical workstation	0.10 pM–10 nM; 0.05 pM–10 nM	60 fM; 10 fM	NR	Milk/ livestock wastewater	[132]
	Tetracycline	Aptasensor	Spectrophotometer	15–500 μM	0.1 μM	120	Milk/raw Beef	[133]

## Data Availability

Not applicable.
